# Extracellular adenosine signaling induces CX3CL1 expression in the brain to promote experimental autoimmune encephalomyelitis

**DOI:** 10.1186/1742-2094-9-193

**Published:** 2012-08-10

**Authors:** Jeffrey H Mills, Leah M Alabanza, Deeqa A Mahamed, Margaret S Bynoe

**Affiliations:** 1 Department of Microbiology and Immunology, College of Veterinary Medicine, Cornell University, Ithaca, NY, 14853, USA

**Keywords:** Extracellular adenosine, CD73, A2A adenosine receptor, CX3CL1 (fractalkine), Experimental autoimmune encephalomyelitis, Multiple sclerosis, Neuroinflammation, Choroid plexus

## Abstract

**Background:**

Multiple sclerosis and its animal model experimental autoimmune encephalomyelitis (EAE) are debilitating neuroinflammatory diseases mediated by lymphocyte entry into the central nervous system (CNS). While it is not known what triggers lymphocyte entry into the CNS during neuroinflammation, blockade of lymphocyte migration has been shown to be effective in controlling neuroinflammatory diseases. Since we have previously shown that extracellular adenosine is a key mediator of lymphocyte migration into the CNS during EAE progression, we wanted to determine which factors are regulated by adenosine to modulate EAE development.

**Methods:**

We performed a genetic analysis of wild type and CD73−/− (that are unable to produce extracellular adenosine and are protected from EAE development) to identify factors that are both important for EAE development and controlled by extracellular adenosine signaling.

**Results:**

We show that extracellular adenosine triggered lymphocyte migration into the CNS by inducing the expression of the specialized chemokine/adhesion molecule CX3CL1 at the choroid plexus. In wild type mice, CX3CL1 is upregulated in the brain on Day 10 post EAE induction, which corresponds with initial CNS lymphocyte infiltration and the acute stage of EAE. Conversely, mice that cannot synthesize extracellular adenosine (CD73−/− mice) do not upregulate CX3CL1 in the brain following EAE induction and are protected from EAE development and its associated lymphocyte infiltration. Additionally, blockade of the A2A adenosine receptor following EAE induction prevents disease development and the induction of brain CX3CL1 expression. The CX3CL1 induced during EAE is found on the choroid plexus, which is the barrier between the blood and cerebral spinal fluid in the brain and is a prime entry point into the CNS for immune cells. Furthermore, CX3CL1 expression can be induced in the brains of mice and in choroid plexus cell line following A2A adenosine receptor agonist administration. Most importantly, we show that CX3CL1 blockade protects against EAE development and inhibits lymphocyte entry into the CNS.

**Conclusions:**

We conclude that extracellular adenosine is an endogenous modulator of neuroinflammation during EAE that induces CX3CL1 at the choroid plexus to trigger lymphocyte entry into the brain.

## Background

Lymphocyte migration into the central nervous system (CNS) is a highly regulated process. Due the potential harmful side effects of inflammation within the brain, many safeguards have evolved to protect the brain from immune mediated damage. For example, anatomical obstacles, such as the blood brain barrier (BBB), prevent the migration of lymphocytes across cerebral blood vessels [1,2]. Additionally, due to the lack of cell-mediated antigen drainage from the brain (the afferent arm of adaptive immunity), brain derived antigens/cells are prevented from priming and activating peripheral T cells [3]. However, migration of lymphocytes into the CNS can occur in response to cellular damage from trauma, bacterial and viral infections, and autoimmune diseases. Since even a small amount of brain damage can have serious or fatal consequences for an individual, there is a delicate balance between initiating an immune response within the brain and quickly controlling it before bystander damage occurs. Although animal models of neuroinflammation have helped to determine which cell types are involved in the brain’s immune response, what promotes the focused migration of lymphocytes into the CNS during an inflammatory attack is not clearly understood.

There are many diseases associated with inflammation in the CNS (including meningitis, encephalitis, cerebritis and encephalomyelitis, etc.). One of the most common neuroinflammatory disorders is multiple sclerosis (MS) [4], a chronic autoimmune inflammatory disease of the CNS that affects more than 2.5 million people worldwide [5]. In patients with MS, loss of neurological function occurs following the demyelination of axons which is mediated by infiltrating autoreactive immune cells. Typically, most patients suffer from a relapsing remitting form of MS, with the disease progressively becoming more debilitating over time. While MS relapses are commonly associated with increased lymphocyte infiltration into the brain and spinal cord, what triggers relapse is not understood. Interestingly, bacterial and viral infections have been implicated in the initiation and persistence of autoimmune diseases [6], including MS [7-9]. Treatments for MS include curtailing the immune response (mitoxantrone, an immunosuppressant or glatiramer acetate, an immunomodulator), preventing immune cell migration (natalizumab, blocks alpha-4 integrin lymphocyte binding), or inhibiting viral replication (interferon-beta; its effects might also be through immunomodulation) [10,11]. Unfortunately, these treatments only reduce the rate of progression of MS, but do not ameliorate it. Therefore, a better understanding of the events which trigger lymphocyte infiltration into the CNS prior to disease relapse is necessary in order to design treatments to halt disease progression.

One potential trigger for immune cell infiltration into the CNS is cell damage. When cells are damaged, extracellular factors are released to communicate the trauma to surrounding cells. An example of such a factor is ATP. Upon damage to a cell membrane, intracellular ATP is released into the extracellular space [12,13]. This ATP can be hydrolyzed into AMP and then to adenosine by the action of the extracellular enzymes CD39 and CD73, respectively [14]. Nearby cells that express any of the four G-protein coupled adenosine receptor subtypes (A1, A2A, A2B, or A3) can respond to the local damage [14,15]. The type of response following the activation of adenosine receptors depends on the responding cell and the adenosine receptors which it expresses. For example, within the lung increased extracellular adenosine levels during injury have been shown to induce cellular migration to promote repair [16]. However, immune cells are typically inhibited by high local extracellular adenosine levels in order to prevent excessive collateral damage to healthy tissue that can result during an inflammatory response [13,14,17,18]. Therefore, extracellular adenosine is arguably an ideal candidate to regulate inflammation, as it acts as a cell damage signal to promote cell migration to sites of tissue damage to promote repair, while also acting as an immune modulator to regulate the magnitude of the inflammatory response and lessen collateral tissue damage [19].

Extracellular adenosine has also been shown to be involved in mediating neuroinflammatory disease progression, in experimental autoimmune encephalomyelitis (EAE), the animal model of MS [20]. Mice that have been given adenosine receptor antagonists or lack the ability to hydrolyze extracellular adenosine from AMP (CD73−/−) are protected from EAE and exhibit diminished lymphocyte infiltration into the CNS [20], despite the observation that lymphocytes from CD73−/− have a more pro-inflammatory phenotype [20]. CD73 and the A2A adenosine receptor are expressed in the brain on endothelial cells [21,22] and the choroid plexus [19,20]. Interestingly, both CD73 and the A2A adenosine receptor are more highly expressed on the choroid plexus than elsewhere in the CNS. [20], sites known to be permissive to CNS lymphocyte entry [23,24]. We hypothesize that damage in the form of released ATP and its subsequent conversion to adenosine during EAE progression is a danger signal to regulate lymphocyte entry into the CNS.

In this study, we demonstrate that extracellular adenosine regulates lymphocyte migration into the CNS during EAE by modulating chemokine expression at the onset of disease. In particular, extracellular adenosine induces the expression of CX3CL1 in the CNS during EAE. CX3CL1 (also known as fractalkine) is a unique molecule that can act both as an adhesion molecule (when membrane bound) and a chemoattractant (following cleavage) for immune cells that express its cognate receptor CX3CR1 [25]. Interestingly, CX3CL1 has been shown to be increased in the serum of patients with MS and other CNS damage [26-28]. Here we show that CX3CL1 increases during EAE progression and can be inhibited through A2A adenosine receptor antagonist treatment (which protects against EAE). Additionally, A2A adenosine receptor agonists induce CX3CL1 expression in the brain and on the choroid plexus. Anti-CX3CL1 antibody mediated blockade protects against EAE development while also inhibiting cerebral lymphocyte infiltration. These findings suggest that extracellular adenosine regulates EAE progression through the induction of CX3CL1 in the CNS.

## Methods

### Mice

C57BL/6 and CX3CR1-GFP/GFP [29] mice were purchased from The Jackson Laboratories. CD73^−/−^ mice have been previously described [30] and have been backcrossed to C57BL/6. Mice were bred and housed under specific pathogen-free conditions at Cornell University. For adenosine receptor blockade experiments**,** mice were given SCH58261 (1 mg/kg or 5 mg/kg *s.c.*; Tocris) 1 day prior to EAE induction and every 3 days continuing throughout the experiment. For adenosine receptor agonist experiments NECA (5'-N-Ethylcarboxamidoadenosine) and CGS 21680 (Tocris) were each dissolved in DMSO, diluted in PBS to the desired concentration, and injected *i.p.* to mice to be compared to vehicle control (DMSO/PBS) treated mice. For anti-CX3CL1 experiments, mice were given either daily rat anti-CX3CL1 IgG (4 μg/mouse, *i.p.*; R&D Systems) or isotype rat IgG (eBioscience) antibody treatment starting at Day 8 post EAE induction (MOG immunization) until the end of the experiment. All procedures performed on mice were approved by the Cornell Committee for the Humane Use of Animals.

### EAE induction and scoring

EAE was induced as previously described [31]. Briefly, a 1:1 emulsion of MOG_35-55_ peptide (1 mg/ml in PBS) (Anaspec) and complete Freund’s adjuvant (CFA, Sigma) was injected subcutaneously (50 μl) into each flank. Pertussis toxin (PTX, 20 ng) (Biological Laboratories Inc.) was given intravenously (200 μl in PBS) at the time of immunization and again two days later. Mice were scored daily for EAE based on disease symptom severity; 0 = no disease, 0.5 = weak tail (cannot curl tail completely), 1.0 = limp tail (complete inability to move tail), 2 = limp tail and partial hind limb paralysis, 3 = total hind limb paralysis, 4 = both hind limb and fore limb paralysis, 5 = death. Mice with a score of 4 were euthanized.

### Choroid plexus cell culture and migration assay

The mouse choroid plexus cell line, CPLacZ-2 [32], was grown in DMEM/F-12 media supplemented with 10% Fetal Bovine Serum (FBS). CPLacZ-2 cells were grown to confluency and treated with the adenosine receptor A2A agonist CGS21680 for 2 hours and then harvested for RNA extraction. For migration assays, CPLacZ-2 cells were grown to confluency on transwell inserts (8 μm). Cells were then treated with CGS (100 nM) for 24 hours. Meanwhile, migratory lymphocytes isolated from the spleen were activated with con A (10 μg/ml) for 48 hours. Before adding migratory cells (2.5 X 10^6^) in to the upper chamber of the transwell, choroid plexus cells were pre-treated with anti-CX3CL1 (1.5 μg/ml) for 1 hour. Lymphocytes were allowed to migrate overnight with exogenous chemokine (SDF-1) added to bottom chamber to induce migration.

### Quantitative real-time PCR

Using Trizol (Invitrogen), RNA was isolated from both mice and the CPLacZ-2 choroid plexus cell line. For mouse samples, animals were anesthetized and perfused with cold PBS through the left ventricle of the heart. Brains were isolated and half of each brain was homogenized in 2 ml of Trizol using the Omni THQ homogenizer (Omni International, Kennesaw, GA). cDNA was synthesized using a Reverse-iT kit (ABGene). Primers specific for CX3CL1 (Forward: 5’-GTGCTGACCCGAAGGAGAAA-3’, Reverse: 5’- CACCCGCTTCTCAAACTTGC-3’) were used to determine gene expression levels relative to housekeeping gene levels using Kapa Sybr Fast (Kapa Biosystems) run on a BioRad CFX96 real time qPCR system. In some instances (noted with hatched lines), comparisons were made to control samples to determine fold change, with controls values set to 1.0. Melt curve analyses were performed to measure the specificity for each PCR product.

### Flow cytometry

Cells were isolated from the spleen of naïve CX3CR1-GFP/GFP mice and then incubated with ACK buffer (0.15 M NH_4_Cl, 1 mM KHCO_3_, 0.1 mM EDTA, pH 7.3) to lyse red blood cells. Cell suspensions were stained with fluorochrome-conjugated monoclonal antibodies against CD45 (lymphocyte marker, 30-F11), CD4 (RM4-5), CD8 (53–6.7), CD49b [Natural Killer NK marker, DX5], CD11b (H35-17.2), CD11c (N418), F480 (macrophage marker, BM8), and B220 (B-cell marker, RA3-6B2) (purchased from eBioscience and BD Bioscience). Stained cells were acquired on a FACS CantoII (BD Biosciences) and analyzed with FACS Diva software (BD Biosciences).

### Immunostaining

Anesthetized mice were perfused with PBS, the brains isolated, and half of the brain was snap frozen in Tissue Tek-OCT medium. Five micron sections (brains in a sagittal orientation) were affixed to Supefrost/Plus slides (Fisher), fixed in acetone, and stored at −80°C. For immunostaining, slides were thawed, washed in PBS, blocked with Casein (Vector) in normal goat serum (Zymed), and then incubated with either fluorochrome-conjugated or non-conjugated [subsequently stained with a goat-anti rat biotin secondary antibody (Jackson ImmunoResearch)] against CD45 , CD11b, F480, CD4, CD8, CD11b, or CX3CL1 (MAB571, R&D Systems). For fluorescent images, slides were mounted with Vectashield mounting media with DAPI (Vector Laboratories). For brightfield images, slides were stained with a biotin/avidin-HRP complex (Invitrogen), developed with an AEC (aminoethyl carbazole) HRP developing kit (Invitrogen), and counterstained with hemotoxylin. Images were obtained on a Zeiss Axio Imager M1 fluorescent microscope utilizing AxioVision software. Cell counts to determine cellular infiltration were performed on anatomically similar areas in the brain (i.e. cerebellum and hippocampus) and spinal cord fields at 10x magnification in each experimental mouse and then averaged together to determine mean cell infiltration per field. Cell counts were performed by the same person for each stain. For fluorescent images, the same exposure/image capture settings were utilized for images taken within the same fluorescent channel for each sample for each specific antibody stain. Utilizing the AxioVision software, brightness and contrast settings (via AxioVision min/max calculation) were similar among the samples within the same fluorescent channel/antibody stain.

### Statistical analyses

Statistical differences between EAE treatment groups were determined utilizing two-way ANOVA analysis. The Student’s *t*-test was utilized for other comparisons unless stated within the figure legends. Statistics were calculated utilizing GraphPad Prism and Microsoft Excel software. Statistical differences were determined where *P* ≤ 0.05.

## Results

### Extracellular adenosine induces CX3CL1 expression in the brain during EAE

**Figure 1:**
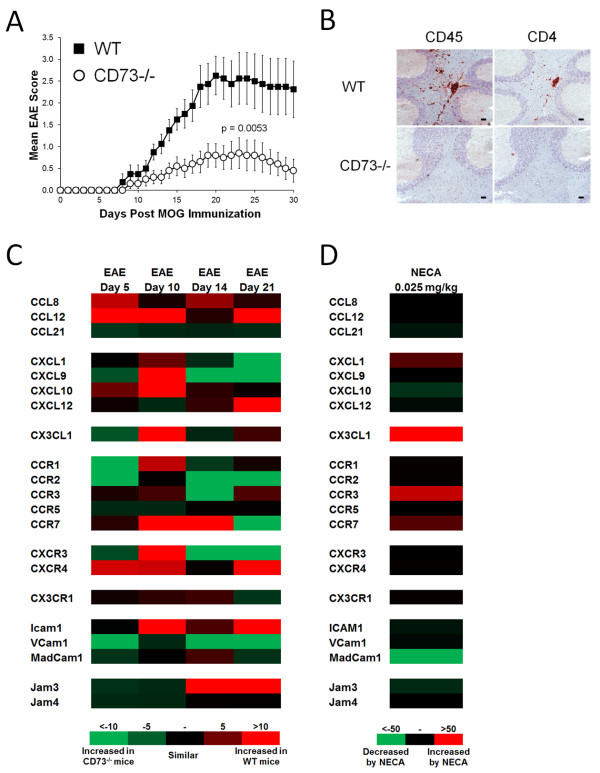
**Chemokine, chemokine receptor, and adhesion molecule genetic expression in the brains of wild type and CD73−/− mice following EAE induction.** (**A**) EAE disease profile of wild type (n = 8) and CD73−/− mice (n = 10). Error bars represent the s.e.m. Significant differences are indicated as determined by two-way ANOVA. EAE data is combined from 2 separate experiments. (**B**) CD45 and CD4 stained frozen brain sections (cerebellum) from day 14 post-EAE induction wild type and CD73−/− mice. Positively stained cells (red) are shown against a hematoxylin counterstain (blue). Black scale bars represent 50 μm. (**C**) Brain gene expression from wild type and CD73−/− mice with EAE over time (n = 5 mice per group) as determined by quantitative real-time PCR. Gene levels were normalized to GAPDH levels and displayed in a heat map as a ratio of wild type to CD73−/− to determine fold differences in expression at each time point. (**D**) Brain gene expression from wild type mice treated with the broad spectrum adenosine receptor agonist NECA (0.25 mg/kg) or DMSO vehicle control as determined by quantitative real-time PCR 4 hours post treatment. Gene levels were normalized to GAPDH levels and displayed in a heat map as a ratio of NECA to vehicle treated to determine fold differences in expression.

Although lymphocyte infiltration into the CNS is required for EAE/MS development, it is not clearly understood what factors regulate this transmigration. Previously, we have shown that extracellular adenosine is a key mediator of lymphocyte migration into the CNS during EAE progression [20]. For instance, mice lacking the ability to hydrolyze extracellular AMP into extracellular adenosine (CD73−/− mice) were protected from myelin oligodendrocyte peptide (MOG) induced EAE development compared to wild type mice (Figure 1A and Table 1) and its associated CNS lymphocyte infiltration (Figure 1B). To identify genes that may be regulated by extracellular adenosine in the brain during EAE progression, we analyzed gene expression of 27 EAE-relevant [33,34] chemokines, chemokine receptors, and adhesion molecules in wild type compared to CD73−/− mice by quantitative real-time PCR (Figure 1C). This detailed study was performed over a time course (Days 5, 10, 14, 21) post-EAE induction. Of the genes analyzed, 21 were differentially expressed at some point during EAE progression (Figure 1C). Of these, CCL12, CXCL9, CXCL10, CX3CL1, CCR1, CCR7, CXCR3, CXCR4, and ICAM1 were increased more than 5 fold in the brains of wild type compared to CD73−/− mice at day 10 post EAE induction (Figure 1C), a critical time point associated with disease onset (Table 1) and initial CNS lymphocyte infiltration [23]. No differences were observed in CCL2, CCL19, CCL20, CXCL2, CXCL11, and CCR6 gene levels (data not shown). 

**Table 1:** **CD73−/− mice develop less severe EAE compared to WT mice**^**A**^

	**Incidence**^**B**^	**Mean Day of Onset**^**C**^	**Mean Max EAE Score**^**D**^
**WT**	8/8	10.8 (8 – 14)	2.9 (1.5 – 5.0)
**CD73−/−**	6/10	12.7 (9 – 17)	1.1 (0.0 – 2.0) *

A. Wild type and CD73−/− mice were induced to develop EAE and scored daily for EAE severity based on the 5 point scale assessing ascending paralysis.

B. Indicates the number of mice that achieved a score of 0.5 (weak tail) in the experimental group.

C. Indicates the average day of onset for the mice that developed EAE (a score of 0.5) followed by the range of scores in parentheses. Mice that did not develop disease were excluded.

D. Indicated the average of the maximum EAE score for each individual mouse followed by the range of scores in parentheses (* = p < 0.01; Student’s *T*-test).

To determine if the expression of these tested genes could be regulated by extracellular adenosine mediated signaling, we treated wild type mice with NECA, a broad spectrum adenosine receptor agonist, and assessed changes in gene expression 4 hours post-treatment compared to vehicle controls (Figure 1D). Only CXCL1, CX3CL1, CCR3, and CCR7 were upregulated in the brains of wild type mice following NECA treatment, with CX3CL1 showing the highest fold increase compared to vehicle treated wild type mice (Figure 1D). As stated above, CX3CL1 was one of the genes identified as being expressed strikingly higher levels in wild type compared to CD73−/− mice during the EAE progression (Figure 1C). Taken together, these results suggest that adenosine receptor signaling triggered by extracellular adenosine hydrolyzed from AMP by CD73 induces the expression of the chemokine/adhesion molecule CX3CL1 in the brain during EAE progression.

### CX3CL1 expression in the brain is regulated by the A2A adenosine receptor

**Figure 2:**
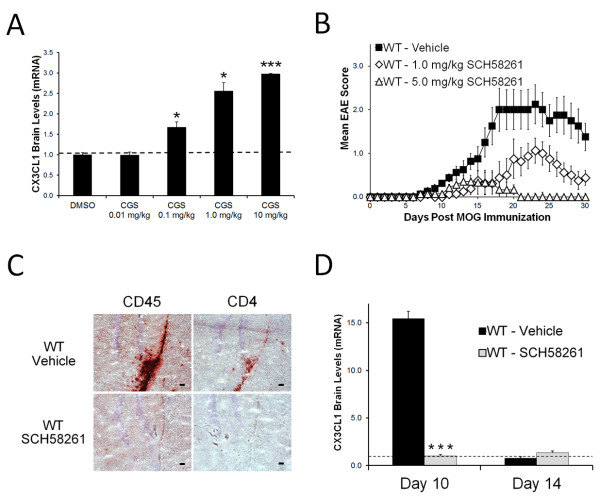
**CX3CL1 upregulation within the brain during EAE is associated with A2A adenosine receptor signaling.** (**A**) CX3CL1 gene expression (relative to DMSO vehicle) in the brains of wild type mice following 4 hour A2A adenosine receptor agonist CGS21680 treatment as determined by quantitative real-time PCR. Error bars represent the s.e.m.; n ≥ 3 mice / treatment. Significant differences (P < 0.05, *; P < 0.001, ***) are indicated as determined by the Student’s *t*-test. (**B**) EAE disease profile in wild type mice given either the A2A adenosine receptor antagonist SCH58261 (1 mg/kg, open diamonds, n = 8; 5 mg/kg, open triangles, n = 7) or a vehicle control (n = 8). Error bars represent the s.e.m. EAE data is combined from 2 separate experiments. (**C**) CD45 and CD4 stained frozen brain sections (hippocampal area) from day 10 post-EAE induction SCH58261 and vehicle treated wild type mice. Positively stained cells (red) are shown against a hematoxylin counterstain (blue). Black scale bars represent 50 μm. (**D**) Brain CX3CL1 expression over time in wild type mice induced to develop EAE and given either SCH58261 or a vehicle control as determined by quantitative real-time PCR. Error bars represent the s.e.m. Significant differences (P < 0.001, ***) are indicated as determined by the Student’s *t*-test.

Previously, we have shown that the A2A adenosine receptor signaling is involved in controlling EAE progression and lymphocyte infiltration into the CNS [20]. To determine if A2A adenosine receptor signaling regulates CX3CL1 expression, we performed dose response studies in mice with the selective A2A adenosine receptor agonist CGS21680 and quantified the gene expression of CX3CL1 in the brain (Figure 2A). Mice that received CGS21680 showed significant increases in brain CX3CL1 expression over a wide range of doses (0.1 mg/kg – 10 mg/kg) compared to vehicle treated controls (Figure 2A). To determine if A2A adenosine receptor signaling regulates CX3CL1 expression during EAE progression, we treated MOG-induced EAE mice with the A2A adenosine receptor specific antagonist SCH58261 (Figure 2B-D). Similar to CD73−/− mice [20], SCH58261 treated mice are protected from EAE development and its associated CNS lymphocyte infiltration (Figure 2B-C). Consistent with this disease protection, genetic analysis of A2A adenosine receptor antagonist treated mice showed no alterations in brain CX3CL1 expression, while vehicle treated mice displayed increased CX3CL1 levels at day 10 post-EAE induction (Figure 2D). These results strongly suggest that A2A adenosine receptor activation, which typically only occurs *in vivo* in environments with high concentrations of extracellular adenosine (such as during inflammation) [35-37], can induce CX3CL1 expression in the brain. 

### CX3CL1 is upregulated in the CNS on the choroid plexus during EAE progression

**Figure 3:**
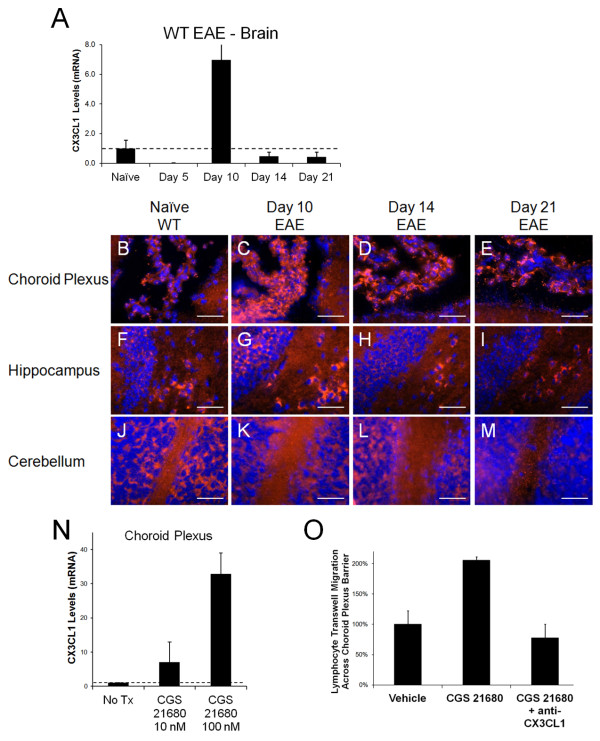
**CX3CL1 expression in the brain during EAE progression.** (**A**) Brain CX3CL1 expression of wild type mice with EAE as determined by quantitative real-time PCR. Gene levels were normalized to GAPDH levels and are displayed as levels relative to naïve mice. Error bars represent the s.e.m. (n = 5 mice per group). (**B**-**M**) Brains from naïve wild type mice were harvested and frozen for immunostaining. CX3CL1 expression (red) and DAPI nuclei staining (blue) in (**B**, **F**, **J**) naïve and (**C**, **G**, **K**) Day 10, (**D**, **H**, **L**) Day 14, and (**E**, **I**, **M**) Day 21 post-EAE induced wild type mice. CX3CL1 expression is displayed at the (**B**-**E**) choroid plexus, (**F**-**I**) in and near the hippocampus, and (**J**-**M**) cerebellum. White scale bars represent 50 μm. (**N**) CX3CL1 expression (relative to non-treated cells) in the CPLacZ-2 mouse choroid plexus cell line after 2 hour treatment with varying concentrations of the A2A adenosine receptor specific adenosine receptor agonist CGS21680. Error bars represent the s.e.m. (**O**) Lymphocyte migration across a transwell choroid plexus barrier following pretreatment with vehicle treatment alone, CGS21680, or CGS21680 and anti-CX3CL1. Total migration was normalized to the vehicle control (set to 100%). Error bars represent the s.e.m. These results are representative of two separate experiments (n ≤ 3).

Because our data indicate that CX3CL1 increased in the brain during EAE progression (Figure 1C and 3A), we next wanted to determine the cerebral structure on which this increase was occurring. Since CX3CL1 is known to be expressed in a membrane bound adhesion molecule form (before it is cleaved into a soluble chemoattractant factor), we assessed its expression via immunofluorescent staining in the brains of naïve and post-EAE induced wild type mice (Figure 3B-M). Based on this staining, CX3CL1 was found to be expressed in naïve mice at low levels on the choroid plexus (Figure 3B) and the hippocampal (Figure 3F) and cerebellar (Figure 3J) areas. At day 10 post EAE induction, when pronounced increases in CX3CL1 mRNA expression is observed (Figure 3A and 2D), increased CX3CL1 expression is displayed at the choroid plexus (Figure 3C), and to a lesser degree near the hippocampus (Figure 3G). CX3CL1 expression at the choroid plexus was still elevated at day 14 post EAE induction (Figure 3D), but returned to naïve levels by day 21 (Figure 3E) when disease recovery was evident. Interestingly, CX3CL1 expression was not found on endothelial cells (Additional file 1: Figure S1). Additionally, no other changes in CX3CL1 expression was observed in the other cerebral compartments during EAE (Figure 3H-I, K-M).

The choroid plexus is located within the ventricles of the brain. It produces cerebral spinal fluid (CSF) and is considered the blood to CSF barrier. Importantly, many reports have indicated the importance of the choroid plexus in both CNS lymphocyte infiltration and EAE progression [23,24,34]. Additionally, the choroid plexus has been shown to express CD73 and the A2A adenosine receptor [20], giving it the capacity to produce and respond to extracellular adenosine. To determine if adenosine receptor activation modulates CX3CL1 on the choroid plexus, we treated the CPLacZ-2 choroid plexus epithelial cell line [32] with varying doses of the A2A adenosine receptor agonist CGS21680 (Figure 3N). Indeed, A2A adenosine receptor activation induced CX3CL1 expression on the choroid plexus cell line 2 hours post treatment (Figure 3N). Furthermore, as CGS 21680 treatment of CPLacZ-2 cells seeded transwells was able to promote lymphocyte transmigration, anti-CX3CL1 antibody treatment effectively inhibited this migration (Figure 3O). In total, these results suggest that CX3CL1 expression on the choroid plexus is modulated by extracellular adenosine to promote lymphocyte migration into the CNS during EAE progression.

### CX3CL1 blockade protects mice from EAE and its associated CNS lymphocyte infiltration

**Figure 4:**
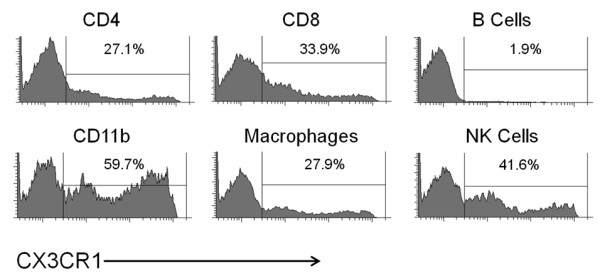
**The CX3CR1 receptor for CX3CL1 is expressed on immune cells.** CX3CR1 expression was assessed on leukocytes isolated from the spleens of naïve mice that express GFP driven by the CX3CR1 promoter (CX3CR1-GFP/GFP mice). Cells were stained with antibodies against CD4, CD8, B220 (B cells), CX11b, F480 (macrophages), and CD49b (NK cells) and analyzed via flow cytometry. The antibody positive gated populations are displayed as histograms with the percentage of CX3CR1 expressing cells within that population noted above each histogram subgate. These results are representative of two separate experiments (n = 4 mice).

Since our data suggests that extracellular adenosine and adenosine receptor signaling regulate CX3CL1 expression to modulate lymphocyte infiltration into the CNS during EAE progression, it is important to determine the importance of CX3CL1 during EAE development. Therefore, we analyzed the expression of CX3CR1 (the receptor for CX3CL1) on immune cells that are known to infiltrate the CNS during EAE and have a role in disease progression. Flow cytometric analysis of CX3CR1-GFP mice [29] showed that CX3CR1 expression was found on subsets of CD4, CD8, CD11b, F480 (macrophages), and CD49b (NK cells) positive cells (Figure 4). CX3CR1 was not expressed on B220 (B cells) positive cells (Figure 4). These results suggest that many immune cells subtypes have the ability to respond to the chemokine/adhesion molecule CX3CL1. 

**Figure 5:**
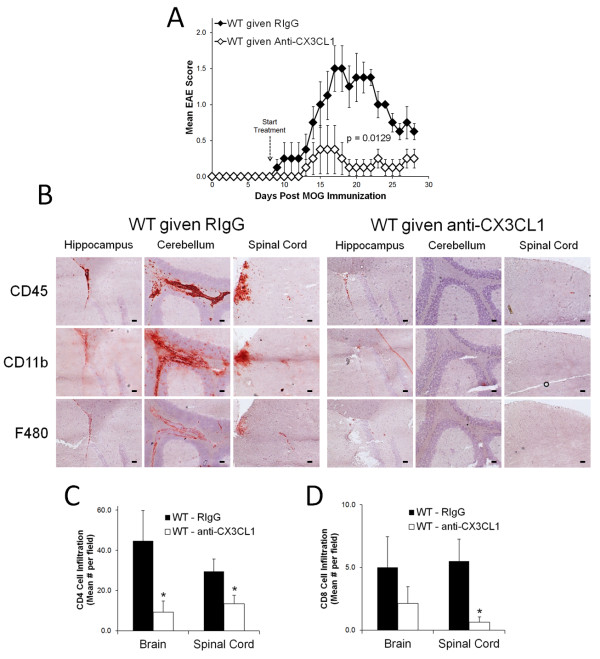
**CX3CL1 antibody mediated blockade protects mice against EAE and its associated lymphocyte infiltration.** Wild type mice were induced to develop EAE and starting at day 8 post induction given daily anti-CX3CL1 antibody or an isotype control treatments (*i.p.*). (**A**) EAE disease profile. Error bars represent the s.e.m. (n = 4 mice/group). Significant differences are indicated as determined by two-way ANOVA. EAE scoring data is representative of 2 separate experiments. (**B**) CD45, CD11b, and F480 stained brain (hippocampal and cerebellum areas) and spinal cord sections from day 28 post-EAE induced mice treated with either anti-CX3CL1 or control antibody. Positively stained cells (red) are shown against a hematoxylin counterstain (blue). Black scale bars represent 50 μm. (**C**) CD4 and (**D**) CD8 positive mean cells counts per field at 10x magnification from brain and spinal cord stained frozen brain sections from day 28 post-EAE induced mice treated with either anti-CX3CL1 or control antibody. Error bars represent the standard error of the mean (n ≤ 11). Significant differences (P < 0.05, *) are indicated as determined by the Student’s *t*-test.

To determine if CX3CL1 activity is important for EAE progression, wild type mice induced to develop EAE were given daily *i.p.* injections of anti-CX3CL1 (neutralizing antibody) or an isotype control antibody starting at Day 8 post-EAE induction (as increases in CX3CL1 are observed during EAE beginning at Day 10) (Figure 5). Based on disease progression, wild type mice that received the anti-CX3CL1 antibody were significantly protected from EAE development, while those that received the control antibody treatment developed significant disease (Figure 5A). Upon examination of the brains and spinal cords from these EAE mice by IHC analysis, we observed fewer immune cells in the CNS of anti-CX3CL1 treated mice compared to control mice (Figure 5B-D). Specifically, while wild type mice that received control antibody treatments showed prominent CD45, CD11b, and F480 positive cell staining in their brain (near the hippocampus and in the cerebellum) and spinal cord, anti-CX3CL1 treated mice displayed minimal staining for the same markers (Figure 5B). Based on cell counts, anti-CX3CL1 treated mice also had significantly fewer CD4 (Figure 5C) and CD8 (Figure 5D) T-cells in their brain and spinal cord (CD4 counts only) compared to control treated mice. It should be noted that no CD49b (NK cell) staining was observed in the brains of anti-CX3CL1 or control treated mice. These results indicate that CX3CL1 plays an important role in the progression of EAE and lymphocyte infiltration into the CNS during disease development. Overall, the data presented here suggest that extracellular adenosine signaling in the CNS induces CX3CL1 expression to induce lymphocyte migration into the CNS to modulate EAE.

## Discussion

The results presented here strongly suggest that extracellular adenosine is an endogenous modulator of lymphocyte migration into the CNS. Because adenosine is an evolutionarily conserved molecule across many species, many innate systems have evolved around the presence of adenosine. Two of these systems are the immune system and the CNS. While extracellular adenosine has a wide variety of effects throughout the body, the fact that it only has a half-life of approximately 6 seconds indicates that extracellular adenosine can only have local effects [38,39]. Here we propose that the presence of high levels of extracellular adenosine within the CNS act as a signal for cellular damage [40,41]. Our data suggest that these high levels are detected by the A2A adenosine receptor expressed at the choroid plexus [20], which induces the expression of the chemokine/adhesion molecule CX3CL1. Subsequent cleavage by ADAM-10 and/or ADAM-17 [42] creates a CX3CL1 concentration gradient from which CX3CR1 expressing cells chemotax to and gain entry into the CNS [43].

While previous findings indicated that extracellular adenosine plays a vital role in neuroinflammation [20], its role in regulating CX3CL1 expression in the CNS was intriguing. Our methodology for detecting genes regulated by extracellular adenosine during neuroinflammation involved 1) identifying genes (previously shown to have a role in EAE development) that were differentially expressed in CD73−/− compared to wild type mice following EAE induction and 2) determining which of these genes were also induced in the CNS of mice following adenosine receptor agonist treatments. Although other genes displayed differential expression patterns between wild type and CD73−/− mice during EAE progression (i.e. CCL12, ICAM-1, CXCL9, CXCL10, CXCL12, etc), CX3CL1 was of interest because it was both upregulated in the brain in wild type but not CD73−/− mice and can be dose-dependently induced in the brain of wild type mice given adenosine receptor agonist treatment. Similar to our findings, other studies have reported that CX3CL1 is involved in EAE and neuroinflammation [26-28,44]. For example, increased CX3CL1 expression was observed in the CNS of rats with EAE in inflammatory lesions [44]. Additionally, elevated CX3CL1 serum levels have been reported in clinical studies in patients with neuroinflammatory diseases, with a positive correlation between CX3CL1 expression and the frequency of lymphocytes in the CSF [27,28]. Furthermore, CX3CR1 expressing cells (which migrate in response to chemotactic CX3CL1 gradients) have been shown to accumulate in brain lesions of MS patients [26]. Overall, these results strongly suggest that CX3CL1 may play a major and complex role in mediating lymphocyte migration into the CNS.

However, the role of CX3CL1 in neuroinflammation is controversial. While the data we present here indicate that CX3CL1 is involved in the triggering events leading to immune cell entry into the CNS, others suggest that it is involved in limiting the degree of inflammation within the brain. These studies describing CX3CL1’s protective role in neuroinflammation utilized the CX3CR1 null mouse and EAE [45,46]. These studies showed that the CX3CR1 null mice develop severe EAE due to a lack of NK cells homing to the CNS [45]. These resident CX3CR1+ NK cells (i.e. present before neuroinflammation) help to limit exacerbated neuroinflammation by interacting with microglial cells and suppressing myelin-reactive Th17 cells [46]. We believe that the results presented in our study do not contradict these previous findings. In fact, since we utilize antibody blockade (which protects against EAE development) and not genetic disruption of the CX3CL1/CX3CR1 axis, our findings further support the idea that CX3CL1 plays an important role in neuroinflammation.

Our study also provides strong evidence that extracellular adenosine is a triggering molecule for inducing lymphocyte entry into the CNS. Previous studies have identified the importance of A1 and A2A adenosine receptors in the progression of EAE [20,47,48]. For instance, mice that lack the A1 adenosine receptor are prone to developing severe EAE [47,48], while mice that receive A2A adenosine receptor antagonists (such as SCH58261) [20,47] are protected against EAE development and its associated lymphocyte infiltration. Interestingly, the A1 and A2A adenosine receptor subtypes are functionally antagonistic to each other and have different affinities for extracellular adenosine [49]. For example, A2A activation (which inhibits adenylate cyclase) only occurs when extracellular adenosine levels reach a higher concentration than required for A1 adenosine receptor activation (which stimulates adenylate cyclase) [35,36]. Since the A1 and A2A adenosine receptors are expressed on the choroid plexus [20], which is highly involved in lymphocyte entry into the CNS [50], neuroinflammation within the CNS may depend on which adenosine receptor is triggered on the choroid plexus. These results suggest that the choroid plexus may act as an adenosine concentration sensor, where high levels of extracellular adenosine (which are produced following cell damage) activate the A2A adenosine receptor and induce lymphocyte infiltration into the CNS.

Despite our results which show the importance of adenosine receptor signaling and CX3CL1 expression at the choroid plexus during EAE, it cannot be overlooked that other areas in the brain may also be involved in regulating neuroinflammation. For example, the blood brain barrier, which consists of astrocytes, pericytes, and endothelial cells, has been shown to be an entry point for immune cells during EAE progression [23,51,52]. Additionally, both astrocytes and endothelial cells have been shown to express adenosine receptors [21,53] suggesting that the blood brain barrier can be influenced by increases in extracellular adenosine levels. Interestingly, since CX3CL1 expression has been observed in astrocytes [44], this suggests that CX3CL1 may be accessible at the blood brain barrier to help promote lymphocyte migration into the CNS during EAE. Additionally, in some instances (such as ischemic brain injuries), adenosine receptor signaling has been shown to be protective against neuroinflammation [54]. Therefore, adenosine’s influence in the brain and on CX3CL1 expression levels during neuroinflammation is a more complex phenomenon than simply described here.

Overall, our study suggests that extracellular adenosine receptor signaling is involved in the development of neuroinflammation. By understanding the underlying mechanisms involved in inducing migration during cerebral inflammation, we show that directed blockade of CX3CL1 can protect against disease development. As cell damage events (i.e. leakage of ATP from compromised membranes) occur in almost all species, the disease protection observed here in mice may be translatable into new therapies for human patients with neuroinflammatory disorders. In fact, drugs used to inhibit lymphocyte migration into the CNS (natalizumab) are currently being used to treat MS [55]. However, the shortcoming of drugs that completely inhibit lymphocyte migration (i.e. through blockade of the alpha-4 integrin) is the consequential induction of temporary immunodeficiency in patients. For example, a well reported potential danger of using natalizumab is the increased risk of progressive multifocal leukoencephalopathy (PML) and other opportunistic infections [55]. Therefore, a better approach in treating neuroinflammatory diseases may be to inhibit certain, but not all, lymphocyte subsets from entering into the CNS (i.e. if MS is caused by a bacterial/viral infection within the CNS). Therefore, by understanding how and why cells are induced to migrate into the CNS, new treatments can be designed for patients with neuroinflammatory disorders. The data presented here mark the continued steps of a journey that will lead to new therapies for MS and other neuroinflammatory diseases.

## Conclusions

We conclude that extracellular adenosine is an endogenous modulator of neuroinflammation during EAE that induces CX3CL1 at the choroid plexus to trigger lymphocyte entry into the brain. This work signifies that adenosinergic agents and the chemokines they induce have the potential to be used in the treatments of neuroinflammatory diseases where pathogenic immune cell entry into the CNS plays a major role.

## Abbreviations

BBB, Blood brain barrier; CFA, Complete Freund’s adjuvant; CNS, Central nervous system; CSF, Cerebral spinal fluid; EAE, Experimental autoimmune encephalomyelitis; MOG, Myelin oligodendrocyte glycoprotein; MS, Multiple sclerosis; NECA, 5'-N-Ethylcarboxamidoadenosine; NK, Natural killer; PML, Progressive multifocal leukoencephalopathy; PTX, Pertussis toxin.

## Competing interests

MSB is a cofounder and holds shares in Adenios Inc. that is developing therapies based on adenosine modulation of CNS barriers.

## Authors’ contributions

JHM and MSB conceived of the study and designed the research plan. JHM (all Figures), LMA (Figures 1, 2, and 3), DAM (Figures 4, 5 and Additional file 1: Figure S1), and MSB (all Figures) performed experiments and analyzed data; JHM and MSB wrote the manuscript. All authors have read and approved the final manuscript.

## Supplementary Material

Additional file 1 **Figure S1.**CX3CL1 is not expressed on endothelial cells during EAE progression in mice. Click here for file
