# Supplementary figures and images for: Extracellular adenosine signaling induces CX3CL1 expression in the brain to promote experimental autoimmune encephalomyelitis
Source: J Neuroinflammation. 2012 Aug 10;9:193. doi: 10.1186/1742-2094-9-193 (PMC3458968; doi:10.1186/1742-2094-9-193)

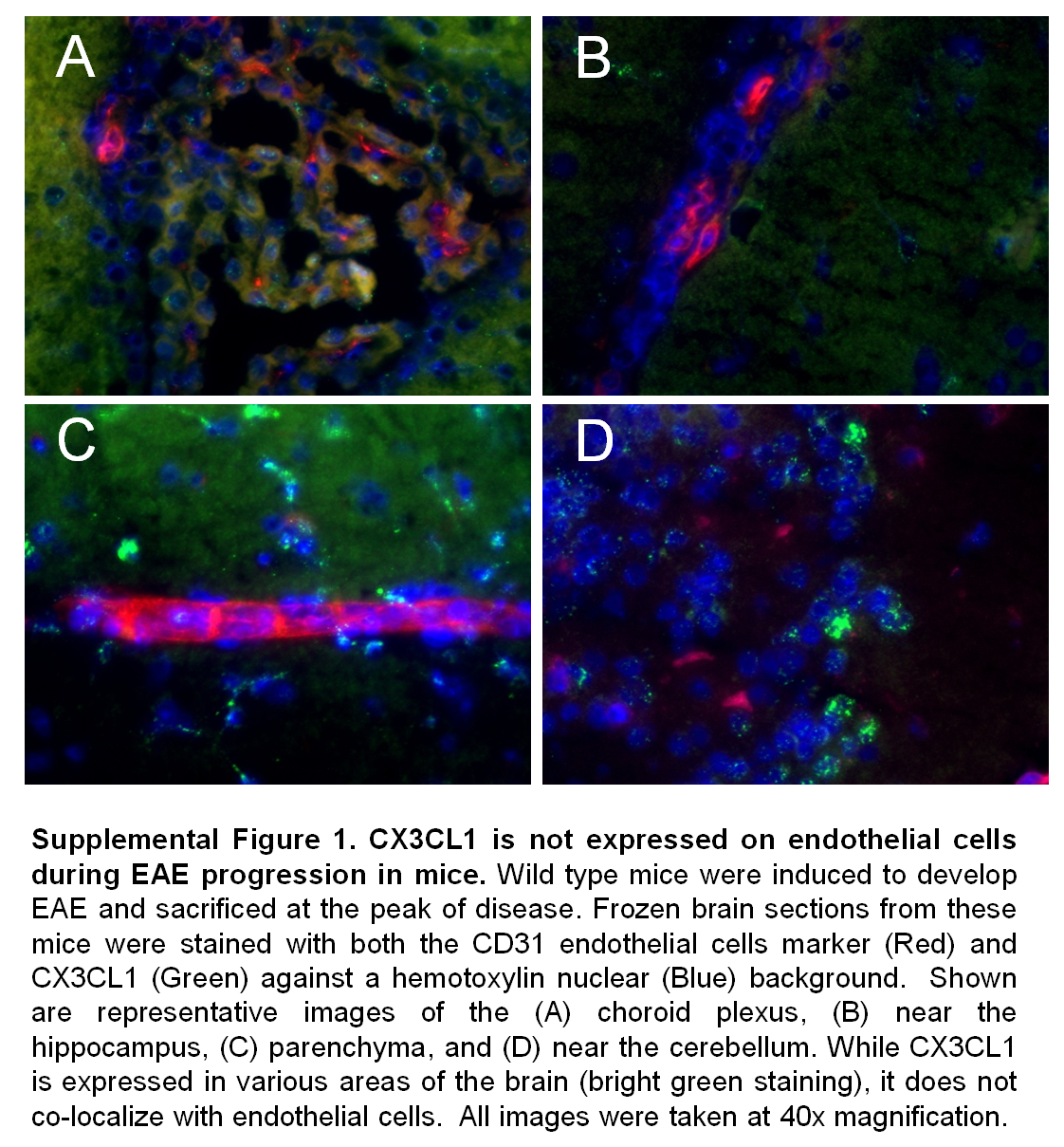

Supplement: Additional file 1 — Figure S1.CX3CL1 is not expressed on endothelial cells during EAE progression in mice. [file 1742-2094-9-193-S1.tiff]
